# Prevalence, Awareness, Treatment, and Control of Hypertension among Young and Middle-Aged Adults: Results from a Community-Based Survey in Rural Tanzania

**DOI:** 10.1155/2020/9032476

**Published:** 2020-09-03

**Authors:** Alfa J. Muhihi, Amani Anaeli, Rose N. M. Mpembeni, Bruno F. Sunguya, Germana Leyna, Deodatus Kakoko, Anna Tengia Kessy, Mary Mwanyika Sando, Marina Njelekela, David P. Urassa

**Affiliations:** ^1^Department of Community Health, Muhimbili University of Health and Allied Sciences, United Nations Road, Upanga, Dar es Salaam, Tanzania; ^2^Africa Academy for Public Health, Plot # 802, Mwai Kibaki Road, Mikocheni, Dar es Salaam, Tanzania; ^3^The Lown Scholars Program, Department of Global Health and Population, Harvard T.H. Chan School of Public Health, Boston, MA, USA; ^4^Department of Development Studies, Muhimbili University of Health and Allied Sciences, United Nations Road, Upanga, Dar es Salaam, Tanzania; ^5^Department of Epidemiology and Biostatistics, Muhimbili University of Health and Allied Sciences, United Nations Road, Upanga, Dar es Salaam, Tanzania; ^6^Department of Behavioral Sciences, Muhimbili University of Health and Allied Sciences, United Nations Road, Upanga, Dar es Salaam, Tanzania; ^7^Department of Physiology, Muhimbili University of Health and Allied Sciences, United Nations Road, Upanga, Dar es Salaam, Tanzania; ^8^Deloitte Consulting Limited, Aris House, Plot # 152, Haile Selassie Road, Oysterbay, Dar es Salaam, Tanzania

## Abstract

**Background:**

Hypertension, which is the single most important risk factor for CVDs, is increasing at an alarming rate in most developing countries. This study estimated the prevalence, awareness, treatment, and control of hypertension among young and middle-aged adults in rural Morogoro, Tanzania. Furthermore, it explored factors associated with both prevalence and awareness of hypertension.

**Methods:**

A cross-sectional survey was conducted as part of the cluster randomized controlled study of community health workers (CHWs) interventions for reduction of blood pressure in a randomly selected sample of young and middle-aged population in rural Morogoro. Sociodemographics, lifestyle-related factors, history of diagnosis, and treatment for hypertension were collected using a questionnaire adopted from the STEPS survey tool. Blood pressure, height, and weight were measured at home following standard procedures. Descriptive statistics were used to estimate prevalence, awareness, treatment, and control of hypertension. Multiple logistic regression models were used to assess determinants of hypertension and awareness.

**Result:**

The prevalence of hypertension was 29.3% (95% CI: 27.7–31.0). Among individuals with hypertension, only 34.3% were aware of their hypertension status. Only around one-third (35.4%) of those who were aware of their hypertension status were currently on antihypertensive medication. Hypertension control was attained in only 29.9% among those on medications. Older age (*p* < 0.001), use of raw table salt (*p* < 0.001), and being overweight/obese (*p* < 0.001) were associated with hypertension. Predictors of awareness of hypertension status were older age, being a female, higher socioeconomic status, use of raw table salt, a history of diabetes, and overweight/obesity (all *p* < 0.001). Alcohol drinking was associated with low awareness for hypertension status (*p* < 0.001).

**Conclusion:**

There is high prevalence of hypertension with low rates of awareness, treatment, and control among young and middle-aged adults in rural Tanzania. Community-level health promotion and screening campaigns for hypertension and other CVD risk factors should be intensified.

## 1. Introduction

Cardiovascular diseases (CVDs) are the leading cause of mortality globally [1]. Hypertension, which is defined as systolic blood pressure (SBP) ≥ 140 mmHg and/or diastolic blood pressure (DBP) ≥ 90 mmHg or regular use of antihypertensive medications [2], is the single most important risk factor for CVDs [3, 4], accounting for 45% of deaths caused by ischemic heart diseases and 51% of deaths caused by cerebrovascular disease [5]. In 2010, the number of people with hypertension was estimated to be 1.4 billion, and with current trend, it is projected to exceed 1.6 billion by 2025 [6].

In 2013, the WHO set a global target for the control of noncommunicable diseases (NCDs), which included a 25% relative reduction in the prevalence of high blood pressure by 2025 [7]. While the prevalence of hypertension declined by 2.6% between 2000 and 2010 in high-income countries (HICs), it increased by 7.7% in low- and middle-income countries (LMICs) during the same period [6]. Similarly, awareness and control for hypertension increased substantially in HICs compared to LMICs [6]. Sub-Saharan Africa (SSA) has the highest rates of hypertension in the world. A study conducted in Soweto, South Africa, reported a prevalence of up to 54.1% [8]. Finding from a systematic review and meta-analysis of studies conducted in SSA reported a pooled prevalence of hypertension of 30%, awareness of 27% among hypertensive individuals, and treatment rates of 18% with only 7% being controlled [9]. In Kenya, findings from a national survey indicated a prevalence of hypertension of 24.5%, awareness of 15.6% among individual with hypertension, 26.9% of those aware were on treatment, and 51.5% were controlled [10].

In Tanzania, available data indicate that CVD risk factors were very low in 1990s [11] but have continued to rise especially among young and middle-aged adults [12, 13]. Data from a national representative STEPS survey indicated the prevalence of hypertension to be 25.9% [14]. A study in Mafia Island in Tanzania reported a high prevalence of 49.5% [15]. Despite the increasing prevalence of hypertension and other CVD risk factors in Tanzania, studies examining awareness, treatment, and control of hypertension are scarce. Both, awareness and treatment for hypertension have been reported to be low in Tanzania. A study in northwestern parts of the country reported awareness and treatment rates of 9.4% and 7.1%, respectively [16]. In contrast, a study in Mafia Island reported higher rates of both awareness (71.2%) and treatment (79.4%) for hypertension [15]. Building on the little existing data, this study assessed the prevalence, awareness, treatment, and control of hypertension and explored their predictors in a random sample of young and middle-aged adults in rural Morogoro.

## 2. Materials and Methods

### 2.1. Study Design and Setting

Data analyzed for this study were collected as a baseline survey for cluster randomized controlled trial of community health workers (CHWs) interventions for reduction of blood pressure. The study was conducted in Kilombero and Ulanga districts in Morogoro region, approximately 450 kilometers southwest of Tanzania's commercial capital of Dar es Salaam. The two districts were selected purposely because of high CVD deaths previously reported in the study area [17].

### 2.2. Study Participants and Eligibility Criteria

Participants were adults aged 25–64 years, residents of the study districts who provided a written informed consent. A resident was defined as an individual who had stayed in the study area for at least 4 months continuously regardless of whether she/he had slept in that household a night before the interview [18]. Household was defined as group of people who served food from the same pot. Self-reported pregnant women and bed-ridden and/or mentally ill health individuals were excluded.

### 2.3. Sample Size Estimation and Sampling Procedures

The sample size for the baseline cross-sectional survey was estimated according to the WHO stepwise approach to chronic disease risk factors surveillance (STEPS) [19]. The sample size was calculated for 95% CI (*z* = 1.96) on the basis of a 5% margin of error, an estimated prior national prevalence of hypertension of 25.9% [14], a design effect of 1.25, and an anticipated nonresponse rate of 10%. Participants were adults aged 25–64 years and categorized into four age-sex groups, resulting into 8 strata. The resulting minimum sample size 3,145.

We used a multistage cluster sampling technique where villages were considered as clusters. A random sample of 12 villages was drawn from a list of 38 villages with active CHWs stratified by district. For each study, village random sample of households was drawn, and at each household, one eligible respondent was selected by a simple random procedure using the next birthday rule. If the selected individual for interview was not available after two home visit attempts, the next eligible member was interviewed.

### 2.4. Data Collection

Data collection was conducted by a team of trained research assistants with experience in conducting health and demographic surveys. A modified WHO STEPS questionnaire for noncommunicable diseases surveillance was used to gather sociodemographic and economic information and behavioral CVD risk factors. Sociodemographic information including age, gender, marital status, education level, and occupation were collected. Age was collected as a continuous variable, while education was measured as the highest level of formal education attained. Marital status was grouped into never married, married or living together, divorced or separated, and widow. Occupation was assessed as a categorical variable as none or housewife, formal employment (public/private), peasant/pastoralist, petty business, and others.

We collected data on household ownership of items such as radio, television, telephone, sofa, refrigerator, bicycle, and car; having working electricity, house ownership, and house construction materials (floor, wall, and roof); source of fuel for cooking and lighting; source of water for drinking and for home use; sanitation facility; and ownership of livestock. A household wealth index in quintiles was then generated following descriptions in the Demographic Health Survey (DHS) toolkit [20] and was used as a proxy for household socioeconomic status.

Behavioral CVD risk factors assessed included smoking, alcohol drinking, and dietary habits. For smoking, questions probed current and past smoking, while for alcohol, they probed current and past alcohol drinking. Dietary assessment included intakes of fruits, vegetables, and use of raw table salt.

### 2.5. Measurements

Blood pressure was measured using a digital blood pressure machine (OMRON HEM-712C, Omron Healthcare, Inc.). Trained research assistants measured blood pressure on the left arm with participant in a seated position. The first reading was taken after at least 5 minutes of resting. The second and third readings were taken halfway and at the end of interview, respectively. An average of three readings was used during analysis. Participants who had elevated blood pressure had a repeat measurement performed on the following day to confirm their elevated blood pressure. Anthropometric measurements included weight and height. Body weight was measured to the nearest 0.1 kg using a SECA 803 digital scale placed on flat ground with participant wearing light clothing and with no shoes. Height was measured to the nearest 0.1 cm with participant in a standing position with heels perpendicular to the portable stadiometer.

### 2.6. Definition of Key Terms

Hypertension was defined as average systolic blood pressure (SBP) ≥ 140 mmHg and/or diastolic blood pressure (DBP) ≥ 90 mmHg or currently taking blood pressure lowering medications in accordance with the Seventh Report of the Joint National Committee on Prevention, Detection, Evaluation and Treatment of High Blood Pressure [2]. Awareness of hypertension was defined as self-report of diagnosis of hypertension among participants who had their blood pressure checked before and responded “YES” to the question “Have you ever been told by the doctor or other health worker that you have hypertension?” Treatment for hypertension was defined as taking blood pressure lowering medications at the time of the survey among those aware of their hypertension status. Control of hypertension was considered for individuals on blood pressure lowering medications if they had systolic blood pressure (SBP) < 140 mmHg and diastolic blood pressure (DBP) < 90 mmHg.

### 2.7. Data Analysis

Data were entered and analysed using IBM-Statistical Package for Social Sciences (SPSS Inc., Chicago, IL, USA) version 20 software for Windows. Data were presented in a frequency distribution with relative frequencies (%) for categorical variables and a measure of central tendency (mean) and of variability (SD) for symmetrically distributed continuous variables. For asymmetrically distributed continuous variables, median with interquartile range (IQR) was used to describe the data. Comparisons between groups were performed using the chi-squared test and independent samples *t*-test (or ANOVA) for categorical and continuous variables, respectively.

Binary logistic regression was performed to examine the relationship of each independent variable with hypertension and awareness of hypertension status. Predictor variables were age, sex, wealth status, marital status, education level, place of residence, occupation, smoking status, alcohol drinking status, use of raw table salt, fruits and vegetable consumption, and body mass index. Variables with *p* < 0.2 in bivariate logistic regression analysis were retained in the multiple logistic regression analysis. Selection of the best predicting model was performed conducting forward, backward, and stepwise model selection procedures. Overall fitness of the model was assessed using the Pearson chi-squared test and Hosmer–Lemeshow goodness-of-fit test. We first assessed the correlation and strength of correlation between variables in the regression model, using variance inflation factor (VIF) with a cutoff value of ≤5 to avoid problem with multicollinearity. Adjusted odds ratio (AOR) and their corresponding 95% confidence intervals (95% CI) are presented as measures of association. Statistical significance was considered based on a two-sided *p* value ≤ 0.05.

## 3. Results

### 3.1. Characteristics of Study Participants

Among 3145 individuals who were approached for participation, 3000 (95.4%) had complete data. Characteristics of the study participants are summarized in Table 1. The median (interquartile range (IQR)) age of the participants was 39 [21–38] years and was significantly higher among men. Higher proportion of the participants (35.7%) was of younger age (25–34 years), while 13.7% was of older age (55–64 years). Majority of the participants were women (74.1%), were married (71.5%), and had primary education (80.4%). Peasant/pastoralist was the predominant occupation (92.5%). Significant sex differences were also observed for marital status, education level, and occupation.

Only 5.9% of the participants was current smokers, and 19.7% was current alcohol drinkers. Both smoking and alcohol drinking were significantly higher in men (all *p* < 0.001). Majority of the participants (85.2%) reported to have ever used raw table salt, with 6.8% reporting to do so always. Men reported to use raw table salt more frequently than women (*p*=0.026). The proportion of participants who consumed vegetables and fruits 5–7 days/week was 63.9% and 7.9%, respectively. Women consumed vegetables more frequent than men (*p* < 0.001). The proportion of participants who were overweight and obese was 28.5% and 16.3%, respectively, and both were significantly higher in women (*p* < 0.001).

### 3.2. Blood Pressure Profile

**Figure 1:**
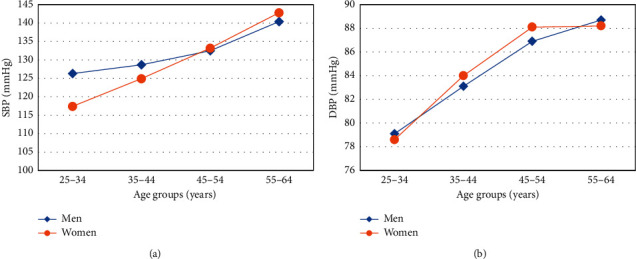
Trend of systolic and diastolic blood pressure by age and sex.

**Figure 2:**
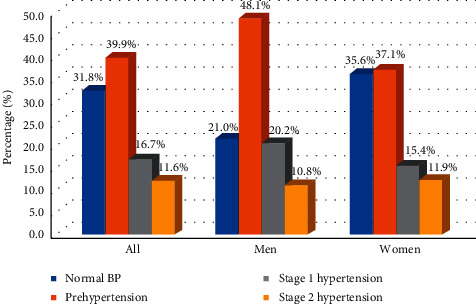
Distribution of blood pressure by sex.

Distribution of blood pressures of study participants is summarized in Table 1. The mean systolic blood pressure (SBP) was 127.2 ± 20.8 mmHg and that of diastolic blood pressure (DBP) was 83.5 ± 12.6 mmHg. Mean SBP was significantly higher among men compared to women (*p* < 0.001). However, no differences were observed for mean DBP. Both SBP and DBP were higher among older compared to young participants. There was a linear increase in SBP and DBP with age in both men and women, although DBP seemed to plateau at 45 years of age (Figure 1). About one-third (31.8%) had normal blood pressure, 39.9% had blood pressure in the prehypertension range, 16.7% had stage 1 hypertension, and 11.6% had stage 2 hypertension (Figure 2). The proportion of participants with prehypertension and stage 1 hypertension was higher in men compared to women (*p* < 0.001).

### 3.3. Prevalence, Awareness, Treatment, and Control for Hypertension

The prevalence of hypertension was 29.3% (Table 2) and increased sharply with age from 12.5% among 25–34 years old age group to 53.2% among 55–64 years groups, respectively (*p* < 0.001). Hypertension was higher among separated/divorced/widowed (*p* < 0.001), among smokers (*p*=0.052), among current alcohol drinkers (*p*=0.022), and among overweight and obese participants (*p* < 0.001). Participants who always used raw table salt had significantly higher rates of hypertension compared to those who never used (*p* < 0.001).

**Figure 3:**
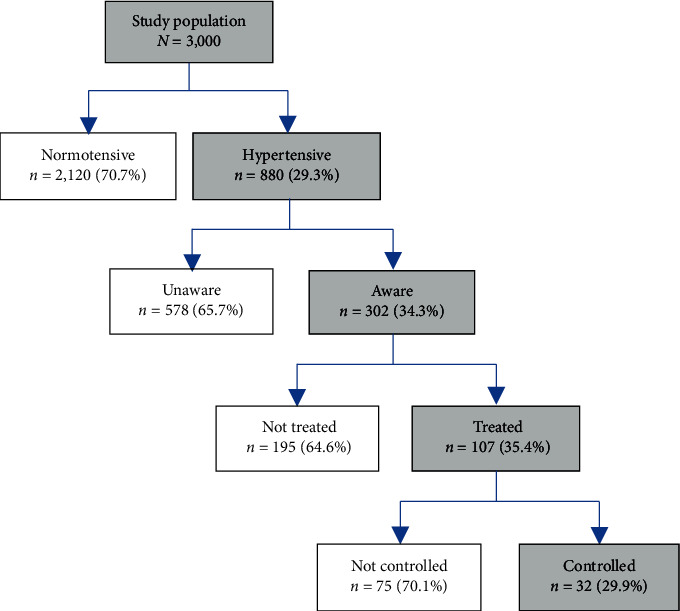
Prevalence, awareness, treatment, and control of hypertension among young and middle-aged population in Morogoro, Tanzania.

Among 880 participants with hypertension, 302 (34.3%) were aware of their hypertension status, while the remaining 578 (65.7%) were newly diagnosed. Of the 302 participants aware of their hypertension status, only 107 (35.4%) were on blood pressure lowering medication, of whom 32 (29.9%) had controlled blood pressure (Figure 3).

Awareness of hypertension was significantly higher among older compared to young participants (*p*=0.016), women compared to men (*p* < 0.001), those with high socioeconomic status (*p* < 0.01), and those with a history of diabetes mellitus (*p* < 0.01). Awareness was low among alcohol drinkers and participants with normal BMI (both *p* < 0.01). Treatment for hypertension was again higher among older compared to younger participants (*p* < 0.001), individuals who used raw table salt, those who consumed fruits more frequently (all *p* < 0.01), and those with a history of diabetes mellitus (*p* < 0.05). Blood pressure control was higher for women, individuals with low socioeconomic status, and those who never used raw table salt. However, none of the factors associated with hypertension control attained a statistical significance level.

### 3.4. Predictors of Prevalence and Awareness of Hypertension

Significant predictors of hypertension in multivariate analysis were older age, use of raw table salt, and higher BMI (Table 3). Compared to young, older participants were 8 times more likely to be hypertensive (AOR = 8.45, 95% CI: 6.33–11.30, *p* < 0.001). Participants who reported using raw table salt had more than twice the odds of being hypertensive (AOR = 2.27, 95% CI: 1.73–2.99, *p* < 0.001). Overweight participants had 53% increased risk (AOR = 1.53, 95% CI: 1.26–1.88, *p* < 0.001), while obese participants had more than double the risk for hypertension (AOR = 2.58, 95% 2.04–3.28, *p* < 0.001).

As for awareness of hypertension (Table 4), predicting factors included older age and being female. Older participants were more than 2 times likely to be aware of their hypertension compared to younger participants (AOR = 2.05, 95% CI: 1.24–3.39, *p* < 0.001). Similarly, women had more than twice the odds of being aware of hypertension compared to men (AOR = 2.47, 95% CI: 1.67–3.66, *p* < 0.001). Other significant predictors of awareness of hypertension were higher socioeconomic status, alcohol drinking, use of raw table salt, history of diabetes mellitus, and higher BMI. Overweight and obese participants were more than 2 times more likely to be aware of their hypertension compared to normal weight participants. Alcohol drinking was associated with 51% decreased odds of being aware of hypertension status (AOR = 0.49, 95% CI: 0.32–0.74, *p*=0.001).

## 4. Discussion

This study provides information on prevalence, awareness, treatment, and control of hypertension and their correlates among young and middle-aged adults in rural Tanzania. Key findings were high prevalence of hypertension, low levels of awareness and treatment rates, and poor control among those on treatment. High prevalence of hypertension was driven by older age, use of raw table salt, and higher BMI. Significant predictors of awareness of hypertension were older age, being female, wealth status, drinking alcohol, use of raw table salt, a history of diabetes mellitus, and higher BMI.

### 4.1. Prevalence and Determinants of Hypertension

The prevalence of hypertension of 29.3% found in our study was comparable to previous report from a community-based survey in Northern Tanzania [39], which found a prevalence of 28.0%. However, it was higher compared to another community-based study in rural Mwanza [16], which reported a prevalence of 8% and that of 25.9% reported in the national representative STEPS survey conducted in 2012 [14]. Low prevalence of hypertension reported in rural Mwanza may be attributable to inclusion of younger participants as more than one-third (38.9%) were aged 15–24 years. Although reports from rural Mafia and Hai districts in Tanzania [15, 40] found higher prevalence of hypertension than observed in our study, the study in Hai district comprised of participants aged 70 years and above in whom hypertension is not uncommon. Overall, two-fifths (39.9%) of participants had prehypertension. While primary prevention strategies should target the general population, more importantly should focus at prehypertensive individuals who are at increased risk of developing hypertension.

In our study, hypertension increased with age ranging from 12.5% among 25–34 years old to 53.2% among those aged 55–64 years. After controlling for other factors, participants aged 55–64 years were eight times more likely to be hypertensive compared to their counterparts aged 25–34 years. Other studies have reported a similar trend [10, 41, 42]. Changes in arteriolar stiffness and elasticity and decreased ability to respond to abrupt hemodynamic changes [43, 44] that occur with advancing age have been implicated for the rise in blood pressure in elderly. Furthermore, hypertension was higher among separated/divorced, and/or widowed, which is consistent with reports from other studies [45, 46]. Marriage has been shown to be protective against CVDs [47]. Marital disruption on the other hand is associated with increased risk for CVDs and mortality in middle-aged women [21, 22], probably due to psychosocial stress [23]. We found lowest prevalence of hypertension among never married, which may be attributable to their young age.

In our study, 44.8% of the participants was either overweight or obese. There is need to increase awareness on the importance of optimal body weight, physical activity, and healthy dietary habits given that the main staple food in the area is white rice. Consistent with findings from other studies [10, 24, 25], we found positive association between overweight and obesity with hypertension. Similarly, a study conducted among Kenyan women was also reported. These findings suggest the need for healthcare providers to prioritize screening for hypertension to overweight and obese clients that come in contact with the healthcare system. Our findings of association between alcohol drinking and smoking with hypertension were also consistent with report from other studies [10, 26].

Use of raw table salt was associated with more than twice increased odds of being hypertensive. High-salt diet leads to the increase in plasma volume and consequently an increase in blood pressure [27]. There is still debate regarding the exact mechanism through which salt intake leads to hypertension [28, 29]. One hypothesis is that increase in plasma volume and cardiac output results from dysfunctional handling of sodium by kidney [28]. Another is through systemic and renal vasodysfunction that salt-sensitive individuals are unable to appropriately decrease systemic vascular resistance in response to increased sodium intake [29]. A higher proportion of Tanzanian has already been shown to be salt-sensitive (46.2%) compared to Brazilians (36.4%) and Japanese (16.7%) and reported tight positive relation of salt with hypertension among Tanzanians [30].

### 4.2. Awareness, Treatment, and Control of Hypertension

Only one-third (34.3%) of hypertensive participants were aware of their hypertension, while the remaining two-thirds (65.7%) were undiagnosed. Despite being low, the rate of awareness in our study is higher than 9.4% reported in rural Mwanza [16] and pooled awareness level of 27% in SSA [9]. High proportion of undiagnosed hypertension can be attributable to unpreparedness of the healthcare system especially lower facilities in rural areas [31]. As a result, most individuals miss opportunity to be screened for hypertension despite their frequent contact with health facilities.

Awareness of hypertension was higher among older participants, women, individuals of higher socioeconomic status, overweight and obese participants, and individuals with a history of diabetes mellitus. Other studies in SSA have also reported higher rates of awareness of hypertension among older individuals and women [10, 32, 33]. Their higher awareness is proposed to be due to their higher contact with the healthcare system. Women privileged in that antenatal care services provide an opportunity for their health check including blood pressure [34]. Similarly, individuals of higher socioeconomic status have higher access to health care services compared to poor. Individuals who reported to drink alcohol had lower levels of awareness of their hypertension. These findings are consistent with reports from other studies [10, 35].

Surprisingly, individuals who reported using raw table salt had higher levels of awareness of their hypertension status, indicating lack of knowledge regarding healthy lifestyles for prevention and control of hypertension in this rural Tanzanian population. Overall shortage of healthcare personnel [36], and more importantly, lack of trained staff for provision of hypertension services at lower health facilities [31], is the underlying reason for continued unhealthy behaviors among hypertensive individuals. Thus, efforts to prevent and control hypertension should ensure enabling lower health facilities to impart knowledge about risk factors, conduct routine screening, and proper management of hypertension.

Treatment among individual who were aware of their hypertension was 35.4%, which is lower compared to other reports in Tanzania [15–17]. Treatment rate among hypertensive individuals regardless of their awareness status was only 12.2%, which is also lower than the pooled treatment rate of 18.0% from systematic review of studies conducted in SSA [9]. Treatment rates were higher among older and diabetic individuals and those who used raw table salt. Rachel et al. found main reasons for not being on treatment to be lack of symptoms, not being prescribed, and finishing a single course of treatment [37].

Less than one-third of study participants on treatment had controlled blood pressure. In contrast, other studies in Tanzania have reported much lower hypertension control rates than found in this study [15, 37, 39]. Blood pressure control has been reported to be higher among individuals with health insurance [38, 48]. Although, the two study districts have good coverage of community health fund (CHF), which offers limited health benefit package to primary healthcare level, we did not assess ownership of CHF cards to draw its associations with hypertension treatment and control. Nonetheless, regular clinic attendance alone cannot guarantee blood pressure control [49]. Lack of trained health personnel and unavailability or frequent stock out of antihypertensive medications are health system challenges facing treatment and control of hypertension in rural Tanzanian settings [31, 50]. As such, community-based primary prevention strategies are the best buys in such settings where access and quality of healthcare services is still low.

This study has both strengths and limitations. Key strengths of this study are large sample size and repeat blood pressure measurements to confirm high blood pressure. Use of three measurements taken on a single session to define hypertension is likely to overestimate the prevalence of hypertension by 5.4%–23.4% [51, 52]. Thus, our study provides a better estimate on the burden of hypertension in this population. Limitations to our analysis include its cross-sectional design, which does not allow drawing conclusions on causal associations between hypertension and independent variables. The sampled population may not be truly representative of the Tanzanian rural population, and these finding should be interpreted with caution. Moreover, we did not collect detailed information of dietary intake using food frequency questionnaire and physical activity, both of which are known to be associated with hypertension.

## 5. Conclusion

The prevalence of hypertension in this rural Tanzanian population was high. Levels of awareness, treatment, and control were unacceptably low. Older age, marital status, education level, smoking and alcohol use, use of raw table salt, a history of diabetes mellitus, and overweight/obesity were identified to be associated with hypertension. These findings call for screening and prevention strategies to curb this increasing burden of hypertension. Community-level health promotion campaigns should be implemented to increase awareness, treatment, and control rates for hypertension, while ensuring routine screening for hypertension is conducted to all individuals regardless of their presenting complaints. Priority should be given to older and overweight and or obese individuals due to their increased risk for hypertension. Healthy lifestyle changes should be encouraged for prehypertensive individuals to halt their progression to full blown hypertension. If implemented fully, such strategies would ensure prevention and control not only of hypertension but also other CVD risk factors.

## Figures and Tables

**Table 1:** Characteristics of the study participants.

Variable	All participants *N* = 3,000 *n* (%)	Males *N* = 778 *n* (%)	Females *N* = 2,222 *n* (%)	*p* value
Age (years) (median (IQR))	39 (31.0–48.0)	41 (32.0–51.0)	38 (30.8–47.0)	<0.001
Age (years)				
25–34	1072 (35.7)	238 (30.6)	834 (37.5)	<0.001
35–44	888 (29.6)	208 (26.7)	680 (30.6)	
45–54	628 (20.9)	182 (23.4)	446 (20.1)	
55–64	412 (13.7)	150 (19.3)	262 (11.8)	

Wealth status				
Poorest	694 (23.1)	190 (24.4)	504 (22.7)	0.046
Poor	497 (16.6)	137 (17.6)	360 (16.2)	
Middle	579 (19.3)	150 (19.3)	429 (19.3)	
Rich	630 (21.0)	134 (17.2)	496 (22.3)	
Richest	600 (20.0)	167 (21.5)	433 (19.5)	

District				
Kilombero	1500 (50.0)	344 (44.2)	1156 (52.0)	<0.001
Ulanga	1500 (50.0)	434 (55.8)	1066 (48.0)	

Marital status				
Never married	383 (12.8)	97 (12.5)	286 (12.9)	<0.001
Married	2144 (71.5)	613 (78.8)	1531 (68.9)	
Separated/divorced/widowed	473 (15.8)	68 (8.7)	405 (18.2)	

Education level				
No formal education	297 (9.9)	32 (4.1)	265 (11.9)	<0.001
Primary education	2411 (80.4)	628 (80.7)	1783 (80.2)	
Secondary education	255 (8.5)	99 (12.7)	156 (7.0)	
College/university education	37 (1.2)	19 (2.4)	18 (0.8)	

Occupation				
Farmer	2775 (92.5)	730 (93.8)	2052 (92.3)	<0.001
House wife	79 (2.6)	0 (0.0)	72 (3.2)	
Employed	27 (0.9)	14 (1.8)	13 (0.6)	
Business	80 (2.7)	25 (3.2)	55 (2.5)	
Others	39 (1.3)	9 (1.2)	30 (1.4)	

Current smoking status				
No	2824 (94.1)	634 (81.5)	2190 (98.6)	<0.001
Yes	176 (5.9)	144 (18.5)	32 (1.4)	

Current alcohol drinking status				
No	2409 (80.3)	547 (70.3)	1862 (80.3)	<0.001
Yes	591 (19.7)	231 (29.7)	360 (19.7)	

Use of raw table salt				
Never	446 (14.9)	95 (12.2)	351 (15.8)	0.026
Sometimes	2351 (78.4)	636 (81.7)	1715 (77.2)	
Always	203 (6.8)	47 (6.0)	156 (7.0)	

Fruits consumption/week				
5–7 days	238 (7.9)	63 (8.1)	175 (7.9)	0.814
1–4 days	2168 (72.3)	567 (72.9)	1601 (72.1)	
Never	594 (19.8)	148 (19.0)	446 (20.1)	

Consumption of vegetables/week				
5–7 days	1916 (63.9)	433 (55.7)	1483 (66.7)	<0.001
1–4 days	1043 (34.8)	333 (42.8)	710 (32.0)	
Never	41 (1.4)	12 (1.5)	29 (1.3)	

History of diabetes mellitus				
No	2971 (99.0)	770 (99.0)	2201 (99.1)	0.838
Yes	29 (1.0)	8 (1.0)	21 (0.9)	

Body mass index (BMI (kg/m^2^))				
Underweight	93 (3.1)	30 (3.9)	63 (2.8)	<0.001
Normal	1563 (52.1)	531 (68.3)	1032 (46.4)	
Overweight	855 (28.5)	170 (21.9)	685 (30.8)	
Obese	489 (16.3)	47 (6.0)	442 (19.9)	

Blood pressure (mmHg)				
Mean SBP (SD)	127.2 (20.8)	131.1 (17.9)	125.9 (20.8)	<0.001
Mean DBP (SD)	83.5 (12.6)	83.9 (11.9)	83.3 (12.8)	0.298

Blood pressure profile				
Normal BP	954 (31.8)	163 (21.0)	791 (35.6)	<0.001
Prehypertension	1198 (39.9)	374 (48.1)	824 (37.1)	
High BP (stage 1)	500 (16.7)	157 (20.2)	343 (15.4)	
High BP (stage 2)	348 (11.6)	84 (10.8)	264 (11.9)	

**Table 2:** Prevalence, awareness, treatment, and control of hypertension among study participants by selected population characteristics.

Variable	Hypertension among study participants (*N* = 3000)		Awareness among hypertensive participants (*N* = 880)		Treatment among aware participants (*N* = 302)		Control among participants on treatment (*N* = 107)	
	Hypertensive %	*p* value	Aware %	*p* value	Treated %	*p* value	Controlled %	*p* value
Overall	29.3	—	34.3	—	35.4	—	29.9	—
Age (years)								
25–34	12.5	<0.001	29.1	0.016	20.5	0.001	25.0	0.705
34–44	29.2		28.6		24.3		38.9	
45–54	42.7		37.3		37.0		32.4	
55–64	53.2		40.6		49.4		25.0	

Gender								
Male	31.4	0.149	21.3	<0.001	38.5	0.615	15.0	0.106
Female	28.6		39.3		34.8		33.3	

Wealth status								
Poorest	28.5	0.701	33.8	0.001	28.4	0.446	52.6	0.143
Poor	28.4		24.1		35.3		33.3	
Middle	28.2		28.8		31.9		20.0	
Rich	30.2		36.3		36.5		20.0	
Richest	31.3		45.2		36.5		29.0	

Marital status								
Never married	24.0	0.001	29.3	0.374	33.3	0.097	11.1	0.336
Married	28.9		34.1		32.2		29.4	
Separated/divorced/widowed	35.7		37.9		46.9		36.7	

Education level								
No formal education	34.0	0.002	31.7	0.193	40.6	0.457	38.5	0.772
Primary education	29.7		35.0		35.2		29.5	
Secondary education	20.0		37.3		26.3		20.0	
College/university	35.1		7.7		100.0		0.0	

Place of residence								
Kilombero district	47.6	0.092	38.4	0.014	31.1	0.089	28.0	0.687
Ulanga district	52.4		30.6		40.4		31.6	

Occupation								
Farmer	29.5	0.588	34.5	0.492	35.3	0.566	30.0	0.457
House wife	21.5		35.3		16.7		100.0	
Employed	29.6		37.5		66.7		0.0	
Business	27.5		18.2		25.0		0.0	
Others	33.3		46.2		50.0		33.3	

Current smoking status								
No	28.9	0.052	35.0	0.122	35.3	0.859	29.7	0.850
Yes	35.8		25.4		37.5		33.3	

Current alcohol drinking status								
No	28.4	0.022	37.6	<0.001	37.0	0.183	27.4	0.107
Yes	33.2		23.0		26.6		50.0	

Use of raw table salt								
No	17.7	<0.001	17.7	0.001	21.4	0.262	66.7	0.158
Yes	31.4		36.0		36.1		28.8	

Fruits consumption/week								
5–7 days	26.5	0.598	31.7	0.825	70.0	0.004	28.6	0.534
1–4 days	29.8		34.9		32.6		32.9	
Never	29.5		33.1		34.5		20.0	

Consumption of vegetables/week								
5–7 days	30.5	0.077	33.4	0.116	35.9	0.876	31.4	0.242
1–4 days	25.3		37.4		34.3		25.0	
Never	36.6		13.3		50.0		100.0	

History of diabetes mellitus								
No	29.1	0.008	33.4	<0.001	34.3	0.044	30.3	0.753
Yes	51.7		86.7		61.5		25.0	

Body mass index (BMI (kg/m^2^))								
Underweight	12.9	<0.001	33.3	<0.001	50.0	0.770	50.0	0.937
Normal	25.0		27.2		37.7		30.0	
Overweight	31.9		35.9		35.7		28.6	
Obese	41.9		45.9		31.9		30.0	

**Table 3:** Crude and multivariate adjusted logistic regression models for determinants of hypertension in the study population (*N* = 3000).

Variable	Crude odds ratio, COR (95% CI)	*p*	Adjusted odds ratio, AOR (95% CI)	*p*
Age (years)				
25–34	Ref		Ref	
35–44	2.88 (2.29–3.63)		2.92 (2.29–3.73)	
45–54	5.21 (4.10–6.63)	<0.001	5.45 (4.20–7.07)	<0.001
55–64	7.94 (6.09–10.3)		8.45 (6.33–11.30)	

Gender				
Male	Ref		Ref	
Female	0.88 (0.74–1.05)	0.149	0.89 (0.72–1.10)	0.286

Wealth status				
Poorest	Ref			
Poor	0.99 (0.77–1.28)			
Middle	0.98 (0.77–1.25)	0.701	—	—
Rich	1.08 (0.85–1.37)			
Richest	1.14 (0.90–1.45)			

Marital status				
Never married	Ref		Ref	
Married	1.28 (0.99–1.65)	0.001	0.87 (0.66–1.15)	0.620
Separated/divorced/widowed	1.76 (1.30–2.37)		0.88 (0.63–1.22)	

Education level				
No formal education	Ref		Ref	
Primary education	0.82 (0.63–1.06)		0.97 (0.73–1.28)	0.615
Secondary education	0.48 (0.33–0.72)	0.003	1.06 (0.68–1.63)	
College/university	1.05 (0.51–2.15)		1.59 (0.71–3.53)	

Place of residence				
Kilombero district	Ref		Ref	
Ulanga district	1.15 (0.98 (1.34)	0.092	1.17 (0.98–1.39)	0.079

Occupation				
Farmer	Ref			
Housewife	0.65 (0.38–1.13)			
Employed	1.00 (0.44–2.30)	0.594	—	—
Business	0.90 (0.55–1.49)			
Others	1.19 (0.61–2.33)			

Current smoking status				
No	Ref		Ref	
Yes	1.37 (0.99–1.88)	0.053	1.12 (0.77–1.63)	0.540

Current alcohol drinking status				
No	Ref		Ref	
Yes	1.25 (1.03–1.52)	0.023	0.93 (0.74–1.15)	0.495

Use of raw table salt				
No	Ref		Ref	
Yes	2.12 (1.64–2.75)	<0.001	2.27 (1.73–2.99)	<0.001

Fruits consumption/week				
5–7 days	Ref			
1–4 days	1.16 (0.83–1.63)	0.599	—	—
Never	1.17 (0.86–1.58)			

Consumption of vegetable/week				
5–7 days	Ref		Ref	
1–4 days	1.32 (0.69–2.50)	0.077	1.45 (0.72–2.94)	0.084
Never	0.84 (0.71–0.99)		0.84 (0.70–1.00)	

History of diabetes				
No	Ref		Ref	
Yes	2.61 (1.25–5.43)	0.010	1.53 (0.69–3.35)	0.293

Body mass index (BMI (kg/m^2^))				
Normal	Ref		Ref	
Overweight	1.41 (1.17–1.70)		1.53 (1.26–1.88)	
Obese	2.17 (1.75–2.67)	<0.001	2.58 (2.04–3.28)	<0.001
Underweight	0.45 (0.24–0.82)		0.41 (0.21–0.78)	

**Table 4:** Crude and multivariate adjusted logistic regression models for determinants of awareness of hypertension status among hypertensive individuals (*N* = 880).

Variable	Crude odds ratio, COR (95% CI)	*p* value	Adjusted odds ratio, AOR (95% CI)	*p* value
Age (years)				
25–34	Ref		Ref	
35–44	0.97 (0.62–1.54)		0.92 (0.57–1.51)	
45–54	1.45 (0.93–2.67)	0.016	1.68 (1.04–2.74)	<0.001
55–64	1.67 (1.05–2.64)		2.05 (1.24–3.39)	

Gender				
Male	Ref		Ref	
Female	2.39 (1.69–3.38)	<0.001	2.47 (1.67–3.66)	<0.001

Wealth status				
Poorest	Ref		Ref	
Poor	0.62 (0.38–1.01)		0.59 (0.35–1.00)	
Middle	0.79 (0.51–1.24)	0.001	0.68 (0.42–1.10)	0.003
Rich	112 (0.74–1.69)		0.94 (0.59–1.48)	
Richest	1.61 (1.07–2.43)		1.48 (0.94–2.32)	

Marital status				
Never married	Ref			
Married	1.25 (0.77–2.01)	0.375	—	—
Separated/divorced/widowed	148 (0.85–2.53)			

Education level				
No formal education	Ref			
Primary education	1.16 (0.74–1.81)			
Secondary education	1.28 (0.63–2.59)	0.292	—	—
College/university	0.18 (0.02–1.44)			

Place of residence				
Kilombero district	Ref		Ref	
Ulanga district	0.71 (0.53–0.93)	0.015	0.80 (0.59–1.09)	0.156

Occupation				
Farmer	Ref			
Housewife	1.03 (0.38–2.83)			
Employed	1.14 (0.27–4.80)	0.519	—	—
Business	0.42 (0.14–1.26)			
Others	1.63 (0.54–4.89)			

Current smoking status				
No	Ref		Ref	
Yes	0.63 (0.35–1.13)	0.124	1.51 (0.76–2.97)	0.238

Current alcohol drinking status				
No	Ref		Ref	
Yes	0.49 (0.34–0.71)	<0.001	0.49 (0.32–0.74)	0.001

Use of raw table salt				
No	Ref		Ref	
Yes	2.61 (1.44–4.73)	0.002	3.20 (1.69–6.06)	<0.001

Fruits consumption/week				
5–7 days	Ref			
1–4 days	1.07 (0.57–1.98)	0.825	—	—
Never	1.15 (0.66–2.01)			

Consumption of vegetables/week				
5–7 days	Ref		Ref	
1–4 days	0.31 (0.07–1.37)	0.138	0.35 (0.07–1.64)	0.140
Never	1.19 (0.89–1.60)		1.26 (0.91–1.75)	

History of diabetes				
No	Ref		Ref	
Yes	0.08 (0.02–0.34)	0.001	12.58 (2.61–60.55)	0.002

Body mass index (BMI (kg/m^2^))				
Normal	Ref		Ref	
Overweight	1.50 (1.07–2.09)	<0.001	1.51 (1.05–2.16)	0.025
Obese	2.27 (1.59–3.23)		2.24 (1.44–3.17)	
Underweight	1.34 (0.39–4.54)		2.14 (0.60–7.60)	

## Data Availability

The data used to support the findings of this study are available from the corresponding author upon request.

## Abbreviations

BMI: Body mass index
CHF: Community health fund
CHW: Community health worker
CVDs: Cardiovascular diseases
DBP: Diastolic blood pressure
DHS: Demographic health surveys
HICs: High-income countries
LMICs: Low- and middle-income countries
NCDs: Noncommunicable diseases
SBP: Systolic blood pressure
SSA: Sub-Saharan Africa
WHO: World Health Organization.

## References

[1] Mensah G. A., Roth G. A., Fuster V. (2019). The global burden of cardiovascular diseases and risk factors. *Journal of the American College of Cardiology*.

[2] Chobanian A. V., Bakris G. L., Black H. R. (2003). The Seventh Report of the Joint National Committee on Prevention, Detection, Evaluation, and Treatment of High Blood Pressure. *JAMA*.

[3] Kearney P. M., Whelton M., Reynolds K. (2005). Global burden of hypertension: analysis of worldwide data. *Lancet*.

[4] Danaei G., Lu Y., Singh G. M. (2014). Cardiovascular disease, chronic kidney disease, and diabetes mortality burden of cardio metabolic risk factors from 1980 to 2010: a comparative risk assessment. *Lancet Diabetes Endocrinology*.

[5] Haldar R. N. (2013). Global brief on hypertension: silent killer, global public health crisis. *Indian Journal of Physical Medicine and Rehabilitation*.

[6] Mills K. T., Bundy J. D., Kelly T. N. (2016). Global disparities of hypertension prevalence and control. *Circulation*.

[7] WHO (2017). *NCDs | Know the NCD Targets*.

[8] Gómez-Olivé F. X., Ali S. A., Made F. (2017). Regional and sex differences in the prevalence and awareness of hypertension: an H3Africa AWI-Gen study across 6 sites in sub-saharan africa. *Global Heart*.

[9] Ataklte F., Erqou S., Kaptoge S. (2015). Burden of undiagnosed hypertension in sub-saharan africa: a systematic review and meta-analysis. *Hypertension*.

[10] Mohamed S. F., Mutua M. K., Wamai R. (2018). Prevalence, awareness, treatment and control of hypertension and their determinants: results from a national survey in Kenya. *BMC Public Health*.

[11] Swai A. B., McLarty D. G., Kitange H. M. (1993). Low prevalence of risk factors for coronary heart disease in rural Tanzania. *International Journal of Epidemiology*.

[12] Mayige M., Kagaruki G., Ramaiya K., Swai A. (2011). Non communicable diseases in Tanzania: a call for urgent action. *Tanzania Journal of Health Research*.

[13] Njelekela M. A., Mpembeni R., Muhihi A. (2009). Gender-related differences in the prevalence of cardiovascular disease risk factors and their correlates in urban Tanzania. *BMC Cardiovascular Disorders*.

[14] Kagaruki G., Mary M. (2016). *Tanzania Steps Survey Report: Ministry of Health and Social Welfare and National Institute for Medical Research*.

[15] Muhamedhussein M. S., Nagri Z. I., Manji K. P. (2016). Prevalence, risk factors, awareness, and treatment and control of hypertension in Mafia Island, Tanzania. *International Journal of Hypertension*.

[16] Mosha N. R., Mahande M., Juma A. (2017). Prevalence, awareness and factors associated with hypertension in North West Tanzania. *Global Health Action*.

[17] Narh-Bana S., Chirwa T., Mwanyangala M., Nathan R. (2012). Adult deaths and the future: a cause-specific analysis of adult deaths from a longitudinal study in rural Tanzania 2003–2007. *Tropical Medicine and International Health*.

[18] Beguy D., Elung’ata P., Mberu B. (2015). Health & demographic surveillance system profile: the nairobi urban health and demographic surveillance system (NUHDSS). *International Journal of Epidemiology*.

[19] WHO (2015). *NCDs WHO STEPS Sample Size Calculator and Sampling Spreadsheet*.

[20] Vyas S., Kumaranayake L. (2006). Constructing socio-economic status indices: how to use principal components analysis. *Health Policy and Planning*.

[21] Gardner J., Oswald A. (2004). How is mortality affected by money, marriage, and stress?. *Journal of Health Economics*.

[22] Zhang Z., Hayward M. D. (2006). Gender, the marital life course, and cardiovascular disease in late midlife. *Journal of Marriage and Family*.

[23] Liu M. Y., Li N., Li W. A., Khan H. (2017). Association between psychosocial stress and hypertension: a systematic review and meta-analysis. *Neurological Research*.

[24] da Costa J. S. D., Barcellos F. C., Sclowitz M. L. (2007). Hypertension prevalence and its associated risk factors in adults: a population-based study in Pelotas. *Arquivos Brasileiros de Cardiologia*.

[25] Basu S., Millett C. (2013). Social epidemiology of hypertension in middle-income countries: determinants of prevalence, diagnosis, treatment, and control in the WHO SAGE study. *Hypertension*.

[26] Zatu M. C., Van Rooyen J. P., Kruger A., Schutte A. E. (2016). Alcohol intake, hypertension development and mortality in black South Africans. *European Journal of Preventive Cardiology*.

[27] Frisoli T. M., Schmieder R. E., Grodzicki T., Messerli F. H. (2012). Salt and hypertension: is salt dietary reduction worth the effort?. *American Journal of Medicine*.

[28] Bloch M. J. (2016). Salt-induced hypertension—what do we really know about the mechanism?. *Journal of American Society of Hyperten*.

[29] Morris R. C., Schmidlin O., Sebastian A., Tanaka M., Kurtz T. W. (2016). Vasodys function that involves renal vasodysfunction, not abnormally increased renal retention of sodium, accounts for the initiation of salt-induced hypertension. *Circulation*.

[30] Mtabaji J. P., Moriguchi Y., Nara Y. (1992). Ethnic differences in salt sensitivity: genetic or environmental factors?. *Clinical and Experimental Pharmacology and Physiology*.

[31] Bintabara D., Mpondo B. C. T. (2018). Preparedness of lower-level health facilities and the associated factors for the outpatient primary care of hypertension: evidence from Tanzanian national survey. *PLoS One*.

[32] Goma F. M., Nzala S. H., Babaniyi O. (2011). Prevalence of hypertension and its correlates in Lusaka urban district of Zambia: a population based survey. *International Archives of Medicine*.

[33] Pires J. E., Sebastião Y. V., Langa A. J., Nery S. V. (2013). Hypertension in Northern Angola: prevalence, associated factors, awareness, treatment and control. *BMC Public Health*.

[34] Pereira M., Lunet N., Azevedo A., Barros H. (2009). Differences in prevalence, awareness, treatment and control of hypertension between developing and developed countries. *Journal of Hyperten*.

[35] Sengul S., Akpolat T., Erdem Y. (2016). Changes in hypertension prevalence, awareness, treatment, and control rates in Turkey from 2003 to 2012. *Journal of Hypertension*.

[36] Sirili N., Kiwara A., Nyongole O. (2014). Addressing the human resource for health crisis in Tanzania: the lost in transition syndrome. *Tanzania Journal of Health Research*.

[37] M Zack R., Irema K., Kazonda P. (2016). Determinants of high blood pressure and barriers to diagnosis and treatment in Dar es Salaam, Tanzania. *Journal of Hypertension*.

[38] Oso A. A., defurin A. A., Benneman M. M. (2019). Health insurance status affects hypertension control in a hospital based internal medicine clinic. *International Journal of Cardiology: Hypertension*.

[39] Galson S. W., Staton C. A., Karia F. (2017). Epidemiology of hypertension in Northern Tanzania: a community-based mixed-methods study. *BMJ Open*.

[40] Dewhurst M. J., Dewhurst F., Gray W. K. (2013). The high prevalence of hypertension in rural-dwelling Tanzanian older adults and the disparity between detection, treatment and control: a rule of sixths?. *Journal of Human Hypertension*.

[41] Joffres M., Falaschetti E., Gillespie C. (2013). Hypertension prevalence, awareness, treatment and control in national surveys from England, the USA and Canada, and correlation with stroke and ischaemic heart disease mortality: a cross-sectional study. *BMJ Open*.

[42] Awuah R. B., Anarfi J. K., Agyemang C., Ogedegbe G., De-Graft Aikins A. (2014). Prevalence, awareness, treatment and control of hypertension in urban poor communities in Accra, Ghana. *Journal of Hypertension*.

[43] Pinto E. (2007). Blood pressure and ageing. *Postgraduate Medical Journal*.

[44] Singh J. N., Dhamoon A. S. (2018). *Physiology, Blood Pressure Age Related Changes*.

[45] Schwandt H. M., Coresh J., Hindin M. J. (2010). Marital status, hypertension, coronary heart disease, diabetes, and death among African American women and men: incidence and prevalence in the atherosclerosis risk in communities (ARIC) study participants. *Journal of Family Issues*.

[46] Sanuade O. A., Boatemaa S., Kushitor M. K. (2018). Hypertension prevalence, awareness, treatment and control in Ghanaian population: evidence from the Ghana demographic and health survey. *PLoS One*.

[47] Hilz R., Wagner M. (2018). Marital status, partnership and health behaviour: findings from the German ageing survey (DEAS). *Comparative Population Studies*.

[48] Hendriks M. E., Wit F. W., Roos M. T. (2012). Hypertension in sub-saharan africa: cross-sectional surveys in four rural and urban communities. *PLoS One*.

[49] Maginga J., Guerrero M., Koh E. (2016). Hypertension control and its correlates among adults attending a hypertension clinic in Tanzania. *Journal of Clinical Hypertension*.

[50] Adinan J., Manongi R., Temu G. A. (2019). Preparedness of health facilities in managing hypertension & diabetes mellitus in Kilimanjaro, Tanzania: a cross sectional study. *BMC Health Service Research*.

[51] Price A. J., Crampin A. C., Amberbir A. (2018). Prevalence of obesity, hypertension, and diabetes, and cascade of care in sub-Saharan Africa: a cross-sectional, population-based study in rural and urban Malawi. *Lancet Diabetes and Endocrinology*.

[52] Dzudie A., Kengne A. P., Muna W. F. (2012). Prevalence, awareness, treatment and control of hypertension in a self-selected sub-Saharan African urban population: a cross-sectional study. *BMJ Open*.

